# How did we get here: what are droplets and aerosols and how far do they go? A historical perspective on the transmission of respiratory infectious diseases

**DOI:** 10.1098/rsfs.2021.0049

**Published:** 2021-10-12

**Authors:** K. Randall, E. T. Ewing, L. C. Marr, J. L. Jimenez, L. Bourouiba

**Affiliations:** ^1^ Department of Civil & Environmental Engineering, Virginia Tech, Blacksburg, VA, USA; ^2^ Department of Chemistry and CIRES, University of Colorado, Boulder, CO, USA; ^3^ Fluid Dynamics of Disease Transmission Laboratory, Massachusetts Institute of Technology, Cambridge, MA, USA

**Keywords:** droplets, aerosols, historical, transmission, respiratory diseases, public health

## Abstract

The COVID-19 pandemic has exposed major gaps in our understanding of the transmission of viruses through the air. These gaps slowed recognition of airborne transmission of the disease, contributed to muddled public health policies and impeded clear messaging on how best to slow transmission of COVID-19. In particular, current recommendations have been based on four tenets: (i) respiratory disease transmission routes can be viewed mostly in a binary manner of ‘droplets’ versus ‘aerosols’; (ii) this dichotomy depends on droplet size alone; (iii) the cut-off size between these routes of transmission is 5 µm; and (iv) there is a dichotomy in the distance at which transmission by each route is relevant. Yet, a relationship between these assertions is not supported by current scientific knowledge. Here, we revisit the historical foundation of these notions, and how they became entangled from the 1800s to today, with a complex interplay among various fields of science and medicine. This journey into the past highlights potential solutions for better collaboration and integration of scientific results into practice for building a more resilient society with more sound, far-sighted and effective public health policies.

## 1. Introduction

The COVID-19 pandemic has exposed major gaps in our understanding of respiratory disease transmission through the air. These gaps led to heterogeneous and shifting transmission mitigation policies from governments and public health organizations. What physical distance should be recommended? What PPE should be used by workers in high-risk settings? Should masks be required among the general public? What types of businesses and activities should be curtailed, and which should remain open? Part of the uncertainty around these questions is rooted in the dissonance between the historical definitions of routes of transmission and current understanding of how pathogens are transported through the air. Traditionally, respiratory pathogens are thought to spread through: (i) direct physical contact between people; (ii) indirect contact through contaminated objects called ‘fomites’; (iii) spray of *droplets* onto the mucous membranes, often considered a subcategory of direct contact transmission; and (iv) inhalation of *aerosols*.

During the first few months of the COVID-19 pandemic in early 2020, the World Health Organization (WHO) and other public health agencies downplayed the *airborne* or *aerosol transmission route*, recognizing it as a potential route for transmission *only* during certain medical procedures such as intubation. Thus, N95 respirators were recommended for healthcare workers only during such procedures but not when otherwise interacting with COVID-19 patients [1]. By the end of March 2020, the WHO posted on social media, ‘FACT: COVID-19 is NOT airborne’, and said that stating otherwise was ‘misinformation’ [2]. Meanwhile, the US Centers for Disease Control and Prevention (CDC) declared in March 2020 that SARS-CoV-2 spreads mainly ‘through respiratory droplets produced when an infected person coughs, sneezes, or talks that can land in the mouths or noses of people who are nearby or possibly be inhaled into the lungs’ [3]. The CDC did not begin using the words ‘airborne’ or ‘aerosol’ to describe transmission until October 2020. As we will see, the differentiation between *droplets* and *aerosols* by the WHO is based on an arbitrary cut-off in droplet diameter; particles larger than the cut-off are considered ‘droplets’ and those smaller are considered ‘aerosols’.

Evidence that airborne transmission was important began emerging in Wuhan in late 2019 and early 2020. In particular, once full airborne precautions were implemented in hospitals, the number of healthcare workers who became infected dropped dramatically [4–6]. Reports emerged in early March of SARS-CoV-2 RNA found on air handling vents in hospital rooms [7,8]. Around this time, some began raising the alarm locally and informally across various countries and institutions. A March 2020 *JAMA* article [9] specifically called into question such recommendations, given that they overlook the well-established physics of respiratory emissions, where droplets of all sizes can be carried many metres within a moist and hot turbulent cloud of exhaled breath. The inability of contact tracing to distinguish between the dichotomous classification of ‘droplet’ and ‘aerosol’ routes is evident when one incorporates such physics of exhalations, from breathing, coughing, talking, sneezing or singing. The *JAMA* article called into question the classification of transmission routes that, at the time, categorically asserted for COVID-19 to be ‘droplet’ based. It also called for the need for masking of both healthcare workers and the general public as a result. More importantly, the author and others called into question the *presumed link* between the 1 and 2 m physical distancing rule and the droplet size-based definition of routes of respiratory disease transmission [10,11]. The links between these notions are the focus of this article. They are central to the debates and disagreements that followed [12–14] about the overall recognition of the role of airborne transmission for COVID-19, as raised in July 2020, by 239 scientists [13] in a letter directed to public health agencies.

**Figure 1.**
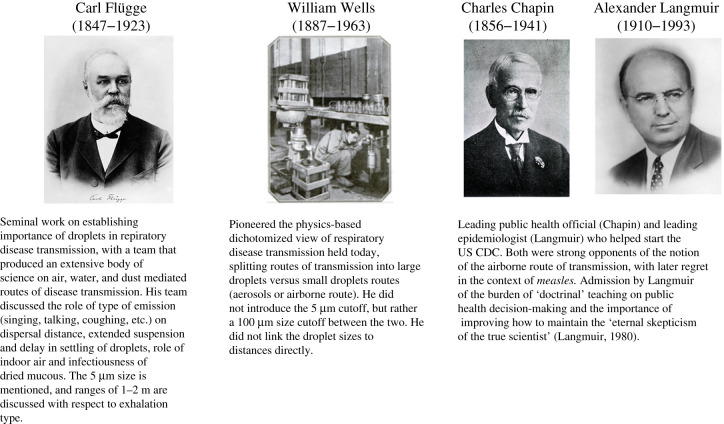
Contributions of key researchers and public health officials in shaping the understanding of respiratory infection transmission and infection control strategies. Mildred Wells and Cretyl Mills, not pictured, also made key contributions to the field.

Throughout these debates, public health agencies have gradually adopted guidance targeting airborne transmission, for example, regarding the need for universal masking as source control [15,16]. However, continued resistance to the role of the airborne route of transmission or misunderstanding of it in general, and for COVID-19 in particular, persists in public health organizations and among leading officials [17,18]. Such disagreements stem at least partly from misunderstandings induced by the very definitions of airborne/aerosol transmission routes. First, *droplet* and *aerosol* transmission are currently defined on the basis of size: ‘droplets’ are considered to be emissions larger than 5 or 10 µm in diameter, whereas those smaller than 5 µm are termed ‘aerosols’. Second, droplets are assumed to follow a semi-ballistic trajectory and to settle within 1–2 m from the person who released them. Yet, these thresholds are not consistent with the physics of droplets and aerosols and the exhalation cloud shaping their transport [9,14,19–21]. To understand how ill-defined nomenclature can potentially hinder scientific and policy progress, it is critical to revisit the historical foundation of these concepts. Only then can we understand how various schools of thought from a range of scientific fields, driven by different trainings and dogmas (e.g. figure 1), have shaped our current knowledge. Historical events and disparate perspectives have also set up roadblocks to aligning fundamental notions of public health and epidemiology with current scientific understanding.

## 2. At the core of epidemics: transmission

The 1918 influenza epidemic provides a useful historical analogy for understanding how a critical public health situation provoked interest in the transmission of infectious diseases. On 14 September 1918, just a few days after the first civilian influenza cases were reported in the USA, a widely circulated warning from Surgeon General Rupert Blue asserted: ‘Mode of transmission—by direct contact or indirect contact through the use of handkerchiefs, common towels, cups, mess wear, or other objects contaminated from fresh secretions. Droplet infection plays an important part’ [22]. In the weeks that followed, a more detailed statement from the US Public Health Service elaborated on the warnings which referred to the mode of transmission in the headline: ‘People Should Guard Against “Droplet Infection”’, with an explanation that included measures to prevent infection: ‘Influenza is always spread from person to person, the germs being carried with the air along with the very small droplets of mucus, expelled by coughing or sneezing, forceful talking, and the like by one who already has the germs of the disease’ [23]. The recommended steps to prevent infection included mask-wearing for all nurses and hospital attendants while near patients, getting fresh air and avoiding crowded spaces, along with this final rhyming reminder: ‘Cover up each cough and sneeze. If you don't, you'll spread disease’ [23].

The similarities in messaging around the COVID-19 pandemic are striking. Dissemination of guidelines from the public health service and syndicated columns in 1918 provided the public with explanations of disease transmission useful in daily life. In 1918, as in 2020, however, these explanations had to strike the right balance between distilling complex and nuanced phenomena, ensuring scientific accuracy and effectively conveying warnings to the general public [24]. In the case of respiratory disease transmission via ‘droplets’ versus ‘aerosols’, this challenge continues to be exacerbated by the fact that scientific understanding of respiratory disease transmission is still evolving, and that fundamental insights are often lost in history, only to be cyclically re-discovered or re-interpreted.

## 3. The swinging pendulum of history: theories of respiratory disease transmission

The mid to late nineteenth century was a transition period in the understanding of infectious disease transmission, public health policy and infection control. The prevailing miasma theory of infectious disease transmission stipulated that diseases were caused by nebulous ‘bad’ air; it did not identify an underlying causative agent and offered little basis for infection control recommendations. While the competing germ theory predated Louis Pasteur and Robert Koch, their careful experiments in the latter part of the nineteenth century helped elevate it from a nascent theory to one with clear and actionable successes. Their scientific approach helped germ theory gain significant traction in the second half of the nineteenth century, as it pushed back against miasma theory [25,26]. With the advent of germ theory, public health officials in the late nineteenth and early twentieth centuries started focusing on hygiene and sanitation practices like handwashing, surface disinfection, waste removal and water purification, which had demonstrable successes for infection control [27]. Yet at the end of the nineteenth century, tuberculosis (TB)—a major public health challenge—remained poorly controlled.

### 3.1. Flügge in the late 1800s

Against this backdrop, Carl Flügge and his assistants in Breslau, Germany, began breaking with the prevailing paradigm that TB was transmitted through the inhalation of fine ‘dust’ of dried tuberculous sputum resuspended in the air from spit bowls, handkerchiefs or other objects. In a series of systematic and extensive experiments Flügge's team demonstrated the existence and efficiency of transmission via fresh exhalations [28,29]. They placed agar plates in rooms at various distances and heights from talking, coughing, singing and sneezing subjects to determine the distribution of contamination in space and time. They also established the infectiousness of the collected samples through transfection experiments on animals. Flügge and co-workers made several key contributions to the understanding of respiratory disease transmission. Initially, their primary focus was not on aerosols versus droplets but about showing that material freshly emitted (sprayed) from the respiratory tract was an important route of transmission *in contrast* with the ‘dry dust’ route. In particular, they emphasized that regular airflows indoors could not easily mechanically break up large volumes of dried sputum into fine dust [28,29]. Second, and contrary to what has later been propagated about their work, they applied the term ‘droplets’ to *all* respiratory emissions, irrespective of the initial droplet size or final constitution (dry or not) *as long* as the emissions *did not* yet settle on surfaces and as long as the emission payload of bacilli was still active. For example, Flügge's team understood that it would take time for respiratory spray emissions to settle, and they waited for up to 5 h following exhalations before removing the plates for analysis. Thus, their results and reference to ‘droplets' encompass both what we now refer to as ‘droplets’ and ‘aerosols/droplet nuclei’ [28–31]. Third, air currents can have a significant influence on the range of the emitted droplets, and so control measures in the context of TB should include not just distancing, but also ventilation, in addition to avoiding crowding [28,29,31]. Fourth, they recognized the notion of pathogen-specific infectious dose to which they linked their recommendations of distancing and duration of exposure in the context of TB [32,33]. Others of the time, such as Koeniger, made clear that recommendations should be pathogen-specific [34]:

…If the pathogenic germs are seldom present in the mouth and in small quantities, as we may assume for the time being from the tubercle bacilli, the danger is only slight in short periods of time with such patients. The probability of an infection then only exists for those people who are constantly in the vicinity of the sick (families, narrow work rooms). But if we were to learn […] that certain types of TB had particularly plentiful bacilli […] as with all other diseases, in which large amounts of pathogens tend to populate the oral fluid, a few coughs, even a few ‘sharp’ words, [would be] enough to completely infect the air of a medium-sized room and give every occupant the opportunity to become infected.

As Flügge's concept of droplet transmission of TB gained acceptance in the early twentieth century, the research, clinical and public health communities appear to have reduced Flügge's encompassing insights to a simplistic interpretation that *only* large visible *liquid droplets* matter in TB. This interpretation was then extrapolated to apply to other respiratory diseases, too. Finally, it was used to claim that airborne routes of transmission had been shown not to be important. This interpretation was also conflated with the idea that only *liquid droplets* that rapidly fall to the ground are involved in close-range transmission. Transmission for respiratory illness would thus only occur over short distances of 1–2 m [35].

Yet, the works of the Flügge team were not so simplistic as portrayed above and by others [36,37]. Flügge emphasized that individual and environmental factors (frequency of coughing and number of droplets shed; number, age and behaviour of people in the vicinity of a patient; poor or cramped housing) need to be taken into account to reduce transmission risk and emphasized the need to keep a distance from *actively* coughing patients [32]. These more nuanced messages seem to have been lost on public health officials, most notably Chapin and Langmuir (figure 1), who forcefully focused only on ‘large’, presumably liquid, droplets, clearly a misrepresentation of the full body of Flügge's work.

### 3.2. Chapin's resistance to the notion of airborne transmission

At the start of the 1900s in the USA, Charles Chapin was a very influential figure in conversations about transmission (figure 1). At the time, Chapin was the Health Officer of Providence, Rhode Island (and later, the president of the American Public Health Association), who by the early twentieth century had gained national recognition in public health circles for his ‘vigorous sanitary action’ [38]. Credited as ‘the foremost teacher of the paramount role of contact infection’ [38], Chapin was convinced that communicable diseases were spread by close contact *only*, including contact with bodily fluids and liquid droplets, and certainly not by transmission of germs through the air. Because of his reputation and the successful implementation of contact-infection-prevention practices in a new hospital in Providence, Chapin's 1910 publication, *The sources and modes of infection*, quickly became a popular guide for public health officials and remained a seminal text for decades [39]. In it, Chapin took a disdainful approach towards the theory of airborne transmission, arguing that evidence did not support such a theory, and that focusing on an airborne transmission route would divert attention from the more relevant contact transmission prevention [27]. The influence of Chapin's doctrine, potentially magnified by a burgeoning public health field that was eager for forward-thinking practices in a post-miasma-theory bacteriological age, kept the focus on short-range contact transmission for the next 20 years.

### 3.3. 1930s Wells' physics-based work on respiratory disease transmission

It was not until the work of William and Mildred Wells in the early 1930s, again in the context of TB, that a systematic science-based approach to understanding respiratory transmission was revived [37]. The Wellses used novel technological advancements in air sampling in addition to physics-based conceptualization and systematic biological experiments. Revisiting the discussion of respiratory emissions, the Wellses introduced a time-scale competition laying the ground for a dichotomous framework of respiratory disease transmission: via *large respiratory droplets* that fall faster than they evaporate, leaving visible stains on agar plates and glass slides in the vicinity of a coughing or sneezing patient; or via *smaller respiratory droplets* that evaporate faster than the time it takes them to settle on a surface, leaving ‘droplet nuclei’ or ‘residues/aerosols’ suspended in the air for potentially long periods of time [40]. The research duo thought of the dried nuclei residues as potentially infectious, and they subsequently became referred to as aerosols in this context. The Wellses did not discuss the distance of fallout of contamination.

It is important to understand the context in which the Wellses' work was ongoing. From an epidemiological perspective, there were consistent and robust observations of ease of transmission in close proximity to infected individuals. Such robust epidemiological observation was, however, and continues to be erroneously interpreted by public health officials and epidemiologists, such as Chapin, to mean that transmission via droplets and fomites is the only relevant route of transmission, and that somehow ease of transmission with proximity discredits aerosol or airborne transmission. Because of this emphasis and association between unrelated concepts—of ease of transmission with proximity to large droplets—the Wellses' assertion that aerosols could be a non-negligible mode of transmission faced resistance. In an early paper, the Wellses note that some rejected a theory of airborne transmission because the term ‘revive[d] the ancient and exploded theory of miasmas’ [37]. They clearly recognized the perception that a theory of airborne transmission could be viewed as *regressive*, re-embracing obsolete ideas of ‘bad air’. In addition to overcoming the erroneous association with miasma theory, another impediment to the acceptance of the Wellses' theory—that infectious aerosols can stay in the air—was the difficulty in establishing conclusive evidence of airborne infection. It was nearly impossible to control an environment well enough to rule out contact (including close-proximity droplet spray) infection.

Experiments from William Wells and his research team in the 1940s regarding air sterilization with UV light seemed promising [41], though subsequent studies failed to reach a satisfying epidemiological conclusion. As a result, many concluded that these failed efforts were because airborne infection was, in fact, not a primary route of transmission. It is surprising that throughout these discussions, the extensive work of Flügge's team seems not to have been revisited. Doing so would have saved time and effort as key concepts, experimental protocols and findings had already been established and tested at that time, which would have supported aspects and nuances of the airborne theory of transmission. Indeed, the Wellses also appear not to have grasped the full extent of Flügge's work, reducing it to ballistic droplets settling quickly as indicated in their quote above [37].

### 3.4. Langmuir and his legacy on the US CDC, and Wells, Riley and Mills

Among the sceptics of the Wellses and those on whom the nuances of Flügge's work appear to have been lost was also Alexander Langmuir (figure 1), the first Chief of Epidemiologic Services at the US Communicable Disease Center (the predecessor organization of the current CDC) until the 1970s. In 1951, Langmuir noted that although ‘[a] large amount of highly suggestive experimental data has been accumulated…, [t]he application of these engineering methods to the control of naturally occurring disease in general population groups […] has been most disappointing. It remains to be proved that airborne infection is an important mode of spread of naturally occurring disease’ [42]. Yet in the late 1950s/early 1960s, the extensive work intellectually led by William Wells, organized by Richard Riley, and conducted by Cretyl Mills was finally published—after the death of Wells—providing definitive evidence of airborne transmission of TB using guinea pigs in the TB wing of a hospital [43]. It is of interest to note that the brunt of this tedious multi-year work was carried forward by Cretyl Mills, who subsequently contracted TB. Wells was the intellectual leader of this research until the very end of his life even while he was suffering from cancer. It is thus surprising that Riley had somehow omitted Wells' name from the final publication to his ‘eternal shame’ as Riley later admitted [44]. We shall thus refer to this publication as Wells, Riley, Mills *et al*. [43] here. From that point on, the airborne theory and Wells' dichotomy of routes of transmission started to become more widely accepted.

Decades after the Wellses' work, Langmuir started to timidly acknowledge that certain diseases were airborne. In the 1980s, Langmuir published a retrospective of his time at the CDC. Given the contemporary understanding of measles as an airborne disease, which he had previously insisted to be transmitted via large droplets/close contact only, he admitted that Wells was right all along [45]. In this pendulum swing of history, it is again interesting to note that in fact, the ‘definitive’ experiment of animal model infection had already been suggested in 1897 [30,46] and subsequently performed by 1899 by the Flügge school, also showing successful infection of animals upon exposure to TB coughing patients [29,47]. The differences were the smaller distance to the patient and less controlled airflow compared to the experiments of Wells, Riley, Mills and co-workers.

## 4. Droplets versus aerosols and origin of the 5 µm threshold

**Figure 2.**
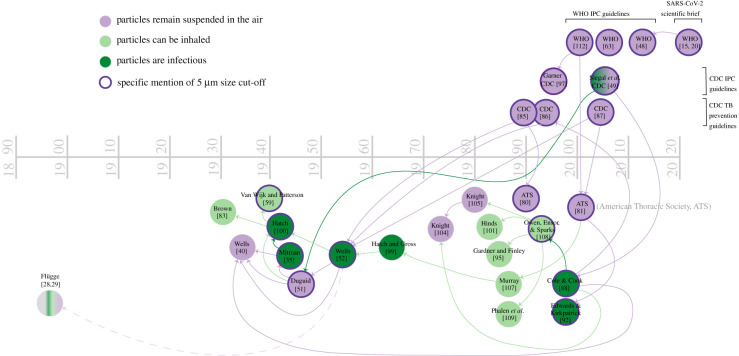
Tracing of how the term ‘airborne’ has been used and understood in the literature, beginning with WHO and CDC definitions as used in infection prevention and control (IPC) guidelines as well as in SARS-CoV-2 scientific briefs. A purple node indicates that a source primarily uses a definition of ‘airborne’ that means ‘particles that remain suspended in the air’. A light green node indicates a source that primarily uses a definition of ‘particles that can be inhaled’, and a dark green node indicates a source that primarily uses a definition of ‘particles that are infectious’. The colour of the arrow connecting the nodes indicates which definition the older source is being used to support in the more contemporary source, even if incorrectly. For example, Siegal *et al.* CDC [49] cite Duguid [51] to support an airborne definition regarding infectiousness, while Duguid [51] understands ‘airborne’ as only what remains suspended in the air. A dotted line indicates the older source is being cited in order to disagree or dismiss the findings of such source, such as Wells [52] and Flügge [28–31].

In contemporary recommendations about droplet transmission, including those regarding COVID-19, both the WHO and the CDC define a distinction between ‘droplets’ and ‘aerosols’ based on a size threshold of 5 µm [15,48,49]. Despite the prominence of this size threshold in the literature [14,50], a 5 µm threshold to distinguish between ‘droplets’ and ‘aerosols’ is not scientifically grounded. Thus, unsurprisingly, the 5 µm cut-off often lacks supporting sources when quoted in the literature, such as in the WHO recommendations [15]. Where citations are provided, they often trace back to William Wells, as shown in figure 2. Yet, Wells' 1934 papers do not mention a 5 µm size threshold. In quiescent ambient air, respiratory particles of this size take approximately 30 min to fall to the ground from a height of 1.5 m (see table 1 in Wells [40], which shows a 10 µm respiratory particle takes 10 min to fall from a height of 2 m and a 1 µm particle takes 16 h to fall the same height). This timescale leaves ample time for transport and inhalation exposure at long distance. In fact, Wells identified the size threshold between droplets that fall to the ground faster than they evaporate versus those that evaporate faster than they settle as 100 µm. To arrive at this number, Wells used a diffusion-based evaporation model in idealized quiescent conditions applied to isolated droplets. The settling timescale is then evaluated using Stokes’ settling speed [52]. However, indoor environments are never quiescent, as temperature and pressure gradients lead to airflow velocities of at least a few centimetres per second. More fundamentally, it has been established over the past 10 years that exhalations in fact contain a continuum of droplet sizes embedded in a turbulent exhalation cloud trapping and transporting them [9,20,21]. Thus, a static fixed size cut-off, particularly in the range of 5 µm, is misleading [19,53].

### 4.1. So where does the 5 µm threshold come from, if not from Wells?

Why do references, particularly in the medical literature or guidelines, often mention 5 µm as the threshold between routes of transmission? In the 1930s, researchers from diverse backgrounds, notably biology and agriculture, began studying microorganisms in the air using new tools and technologies [54], launching the field of aerobiology [55]. An interest in airborne microorganism dispersal sprouted in other disciplines, too, including mycology, dermatology and respiratory occupational health, pertaining, for example, to farmer's lung disease [56]. At the same time, interest in outdoor air quality was also growing. These efforts were focused mostly on the receiving end of the system: the inhalation of contaminants and their potential to deposit in the lungs. Therefore, discussion about particle size revolved mainly around its relationship to the potential for inhalation, penetration and deposition in different regions of the respiratory tract [57], and not on how the particles move through the air. In this general context, industrial hygiene studies from the 1930s and 1940s suggested that only the smallest particles—1–5 µm—could reach the deepest part of the lungs [58,59]. Thus, this size threshold became associated in the subsequent literature by public health and infectious disease researchers with what was thought to be most infectious [49]; see figure 2 for mentions of *inhalation* and *infectiousness* in the displayed references. Interestingly, the older works of the Flügge school appear to have been lost in translation again: they had already discussed ‘bronchial droplets' for the droplets presumed to be most effective in TB infection. They claimed that exhaled droplets in a specific size range contained the largest amounts of TB bacilli [33,47]. In contrast with modern discussions of this concept, they considered bronchial droplets to be 20–60 µm in size. Conversely, Flügge noted difficulty in infection of animals for artificially sprayed droplets that were significantly larger than 40 µm [32].

## 5. Renewed interest in airborne infection: biological warfare

While Wells’ work regarding airborne transmission was labelled as having ‘failed’ the ‘challenge to the theory of contact and droplet infection’ by Langmuir [42], his work was nonetheless considered foundational to understanding the *physics* of airborne infection. In the same 1951 presentation in which he disparaged Wells’ belief that airborne infection could occur naturally, Langmuir [42] acknowledged that ‘the knowledge accumulated during the past 15 years has clearly laid the scientific basis for the mechanisms of airborne infection’ noting that airborne spread was now commonly recognized as a cause for *artificially* induced human infections. Delivered only months after the start of the Korean War, Langmuir's address was intended to prepare public health students for the possibility of airborne infection via biological warfare. This was the motivation for his sudden marriage of epidemiological concern and what he had dismissed in the prior decade regarding airborne transmission. The emerging aerobiology and aerosol science insights built over the previous decades included the recognition that upon inhalation, ‘[p]articles larger than 5 µm in diameter are almost completely removed in the nose and upper respiratory passages’ while ‘progressively increasing proportions of inhaled particles reach the terminal bronchioles and alveoli’ when below 5 µm in size [42]. If airborne transmission was not considered to occur naturally but *was* understood to occur under artificial circumstances, the threat of aerosolized pathogens delivered *en masse* to the American population was a dramatic reason for the epidemiological community to focus again on airborne infection and public health, even if such a concern had been previously dismissed.

Embracing this marriage between epidemiology and aerosol science, William Wells published a book in 1955 [52] that expanded significantly on his theory of airborne transmission. Like Langmuir, Wells now referred to the research that showed particles 1–5 µm in size were small enough to reach deep into the lungs. The more sophisticated technologies developed in the subsequent 20 years seemed to encourage a new focus for Wells: not just what stayed suspended, but what could be truly *infectious* based on the ability to reach the deepest parts of the lungs. Though no association is made explicit in any of the literature we reviewed, this focus on particle deposition in the lungs likely popularized the 5 µm diameter cut-off. A turn *back* towards a theory of airborne infection with TB in the 1960s may have resulted in the entanglement of deposition in the lungs (5 µm cut-off) with Wells’ dichotomized definition of droplet versus airborne transmission (cut-off of 100–200 µm) in infectious disease protocols and public health guidelines. Additionally, in public remarks made in 1964 [60], Langmuir used the 5 µm distinction between *large droplets* and *aerosols*, explicitly stating that droplet size *is* relevant because of where the aerosols are deposited in the lungs.

In sum, tracing the origins of the 5 µm threshold, as cited in public health literature (figure 2) ultimately revealed a conflation between various understandings and definitions of ‘aerosols’. Most contemporary sources use this threshold only to explain which particles stay suspended in the air for longer times, yet the 5 µm distinction is clearly *not* based on what *stays airborne* but on what *reaches deepest in the lungs, irrespective of a pathogen's tropism*. It is this conflation of particle transport through the air and particle deposition in the lungs that appears to be the source of the error in distinguishing between droplet and aerosol transmission routes as defined by a 5 µm threshold. The problems created by this conflation are many. First, it fosters a misunderstanding among health professionals about most infectious particles (such as those carrying SARS-CoV-2) not remaining airborne. Second, it codifies a particle size based on the pathogenesis for TB that research shows does *not* apply to other infectious diseases. Viral receptors for SARS-CoV-2 are located throughout the respiratory tract for example [61], and initiation of infection in the nose and upper respiratory tract is thought to be important [62]. Therefore, unlike for TB, aerosols of sizes all the way up to the inhalable limit of 100 µm are capable of initiating infection. Third, the size of a droplet upon emission is not necessarily the size upon inhalation and is not a size that necessarily remains constant after exhalation and inhalation, due to evaporation and rehydration [20]. If a reference to a specific droplet size needs to be made, a standardized procedure for such measurement is key [20]. A size cut-off and dichotomy are useful for general conceptualization and broad understanding of the route of exposure and control measures. However, a detailed understanding of the droplet size physics, the flow dynamics (in space and time), and their measurement are critical to providing sound scientific underpinning of interventions and to eliminating inconsistencies in public health guidelines and associated false debates.

## 6. Droplet sizes and range: origins of the 1–2 m rule

**Figure 3.**
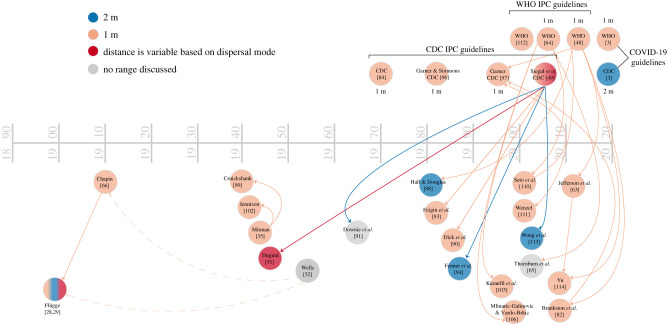
Tracing of citations of standard droplet infection ranges as used by both the WHO and the CDC. Nodes in blue indicate the use of a 6 foot/2 m designation, while nodes in peach indicate the use of a 3 foot/1 m designation. Sources in red argue for a variable range. Arrows between nodes indicate how an older source is cited in the newer. In some cases, the edge colour matches the node colour (e.g. WHO [48] cites Jefferson *et al.* [63] to support a 3 foot range) and in others, it does not (e.g. WHO [64] cites Thornburn *et al.* [65] to support a 3-foot range, but Thornburn *et al.* [65] do not use that distance, as indicated by its grey node colour). A few key sources in the historical droplet/aerosol conversation are included here despite being outside of the citation trace working backward from WHO/CDC guidelines, both to keep some continuity with figure 2 and to show historical shifts in understanding. For instance, Wells [52] is included, with a dotted edge to both Chapin [66] and Flügge [28–31] to indicate disagreement with how both sources understand droplet range.

Alongside the common definition of aerosols as being smaller than 5 µm (figure 2), contemporary guidelines for infection control in healthcare protocols as well as in public health messaging distinguish spray-borne droplet infection from the airborne route by the *distance* travelled by a droplet or aerosol (figure 3). Such distance has been widely used to drive a range of *social/physical distancing rules worldwide*—from 1 to 2 m—also with heightened debates regarding implications for society's basic functioning [11]. Interestingly, the recommended distance to avoid infection varies from 1 m per WHO and in parts of Europe, to 1.5 m in Australia, to 2 m in the USA, Canada and the UK. The most recent CDC Guidelines for Isolation Protocols in Healthcare Settings note that, historically, the accepted distance for ballistic droplet transmission has been within 3 ft [49] (figure 3). They also note the more recent studies from SARS-CoV-1 in the early 2000s that demonstrate an infection distance of up to 2 m or more. Although the 2003 SARS-CoV-1 pandemic never became widespread enough in the USA to warrant non-pharmaceutical interventions, public-facing information about SARS-CoV-1 from the CDC indicated that droplet spread generally happened within 3 ft [67]. Thus, the current pandemic is the first recent implementation of a 6-foot recommendation in the USA, although public health warnings during the 1918 influenza epidemic included social distancing, based on research at the time about droplet infection [68,69]. We found that these short distances have been brought to institutional attention since at least the mid-nineteenth century. During the Crimean War (1853–1856), the Royal Commission recommended keeping beds in soldiers’ barracks at least 3 ft apart, noting that such measures lowered incidences of respiratory illness as might be expected regardless of the route of transmission [70]. Likewise, Biernacki advocated spacing beds a certain distance apart to create an ‘invisible barrier’ to limit a ‘pathogen's excursion’ [71]. While such recommendations are based on correct empirical and epidemiological observation, namely that in general larger distancing reduces the incidence of respiratory disease transmission, the mechanistic justification for such recommendations, linking them *only* to short-range liquid droplet fallout on another person or surfaces to the exclusion of inhalation of aerosols, is erroneous. Hence, the *scientific basis* that inherently links respiratory disease transmission mechanisms to a prescribed distance of 1–2 m requires urgent and careful revisiting.

The first mechanistic investigations seem to date back, again, to Flügge [28,29]. Experiments in the late nineteenth century not only presented evidence of infectious droplets, but also provided material evidence of how far those droplets might travel in unprotected exhalations. In experiments by von Weismayr and by Laschtschenko from the Flügge school, for example, subjects were given a saline suspension of *Serratia marcescens* to rinse their mouths and were then asked to speak, sing, cough and sneeze. The results showed that colonies grew in Petri dishes positioned as far as about 2 m from the speakers, 4 m from the coughers and as far as 9 m from the sneezers [31,72], in the absence of notable background airflows. It is important to recall again that ‘droplets’, in the context of the Flügge school, encompassed both liquid and dry particles of all sizes prior to settling on a surface. This is why, contrary to many other studies that reported no contamination beyond 1–2 m, the Flügge team waited up to 5 h prior to plate collection [28,29,31]. These results were confirmed by Koeniger [34], who additionally allowed for common indoor airflows to persist to increase the realism of the experiments; he noted that ‘when speaking, a transfer of the droplets had taken place up to the extreme corners of the very large room’, up to 12.5 m [34]. Finally, a speaker at the dispatch box of the expansive UK House of Commons also gargled with a broth culture of *Bacillus prodigiosus* before reciting Shakespeare passages in a loud voice to the empty room; although growth colonies were more numerous in plates near the speaker, cultures were apparent on plates over 21 m away [73]. Others followed suit with similar experiments [74]. So, what mechanistic study links historically to the 1–2 m rule? As mentioned above, it is only in the narrow context of TB transmission that Ziesché and Flügge made explicit reference to a distance of about 1 m associated with a specific exposure time linked to an extrapolated threshold of infectious dose of 400 bacilli [32,33]. Any concrete distance recommendation clearly requires revisiting in the context of specific pathogens, environments and infectious doses, as already pointed out by Koeniger in 1900 [34].

The limitations of the plate collection method were emphasized by the Wellses’ observation in the 1930s that small particles would remain airborne without settling at all. Thus, plate collection over a short time was an inappropriate methodology for detection of microorganisms in the air. With the evolution of new measurement tools, particularly photography in the 1940s, it became possible to directly visualize the emissions from sneezing, coughing or talking. Such visualizations, however, were still limited to windows of observations 1–2 m in width, and the smallest particles still could not be detected by optical methods. The lack of imaging beyond the 1–2 m distance [75], combined with prior misrepresentations of Flügge's work and others on plate collections of organisms, reinforced the collective focus on this 1–2 m distance as a primary danger zone.

In recent years, a number of other studies have found organisms and respiratory emissions collected beyond a 1–2 m distance in the context of a range of respiratory diseases [10,11]. Recent findings in fluid dynamics of respiratory emissions also support the view that the framework of ‘droplet’ versus ‘aerosol’ routes of transmission is not a perfect dichotomy with a sharp boundary in particle size and distance and make clear that a 1–2 m distance is not compatible with the physics of respiratory emissions [9–11,21,76].

Given all the above, and that distancing and droplet size cut-off concepts did not originate together, it is puzzling that they continue to be linked. An entrenched belief persists that ballistic droplets are the primary route of infection and that above a particular fixed size, they settle out within 1 or 2 m, when in fact, as explained by Flügge, the concept of distance is linked to an infectious dose that can be reached also by inhalation of droplets of any size that have not yet settled. Such entrenched confusion between these concepts is the root of the neglect of the airborne route of infection in guidelines, despite the mounting casualties of the COVID-19 pandemic. Thus, similar to the recommendations from the late 1800s, guidance continues to be based largely on empirically observed infection mainly among close contacts, rather than a mechanistic understanding of where, how long and in which environments pathogen-laden droplets can remain airborne and infectious.

## 7. Outlook/discussion/conclusion: role of public health organizations—recommendations for changes

As case studies of transmission of COVID-19 continue to accumulate [77], it is increasingly clear that transmission is not accurately represented by the ‘droplet route’ and its erroneous association with the 1–2 m rule [9,10,13]. In fact, the importance of aerosol transmission is becoming obvious from a range of emissions from breathing to talking, to singing, to coughing, to sneezing (see fig. 3b in [19]), with a number of remaining open questions on the nature of their creation and pathogen load upon emission. A more nuanced understanding is required so as to guide also a more nuanced People-Air-Surface-Space (PASS) strategy of management of COVID-19 [11,19,20,78] that advocates an integrated approach to risk mitigation, combining personal protection, air and surface hygiene and space management to reduce the risk of infection.

What we hope to demonstrate in this essay is that although ideas about droplet size (figure 2) and range of spread (figure 3) are seemingly well accepted, their foundation is muddled and misleading, and is not consistent with physics. With the confusion over SARS-CoV-2 transmission that has led to conflicting public health recommendations, this is a crucial moment to clarify and realign infection control guidelines to match both historical and contemporary understandings of airborne transmission, including moving away from using TB as the reference standard of airborne infection.

Though researchers from multiple (and often disconnected) fields are studying airborne transmission from different perspectives, a move towards consistent conversations and research about airborne transmission—instead of ad hoc conversations that arise only in times of crisis—will give researchers, public health officials and infection control specialists the time needed to collaborate, discuss limitations and ultimately better implement science into policy. We call for a model in which this research is continually advanced, particularly between epidemics and pandemics. We also call for the inclusion of a broader range of expertise in key scientific and decision-making committees. For example, the WHO Infection Prevention and Control (IPC) committee contained multiple handwashing experts, but no airborne disease transmission experts, including expertise from aerosols to fluid dynamics, even though the modes of transmission of the new disease were very unclear. Closer and continuous integration of scientific results with policy is also important, with support, expansion and strengthening of initiatives such as training and pairing programmes between researchers and policy translators at the heart of engineering and science focused programmes rather than separate from them (e.g. International Policy Laboratory at MIT [79]). Additionally, closer integration mechanisms between research on academic campuses and at national/federal/global institutions could bridge this gap. This could be achieved through, for example, integrated research and policy translation teams, funding support for such purposes or specialized offices for aiding translation of research results into policing relevant information.

We hope that this closer look into history and revisiting the origin of these concepts, and how such concepts evolved to be adopted *or not* into public policy since the late 1800s, shows that the swinging pendulum of history is at play. Thus, maintaining a historical perspective is key to enable productive communication between various scientific and public health communities aiming to design more sound, resilient, far-sighted and effective public health policies for slowing the transmission of respiratory infections when they emerge.

## Data accessibility

Data will be made accessible upon request.
